# The relationship between spiritual health and happiness in medical students during the COVID-19 outbreak: A survey in southeastern Iran

**DOI:** 10.3389/fpsyg.2022.974697

**Published:** 2022-08-11

**Authors:** Mahdi Abdolkarimi, Mahdieh Masoomi, Seyedeh Shirin Lotfipur, Mohammad Ali Zakeri

**Affiliations:** ^1^Department of Health Education and Health Promotion, School of Health, Rafsanjan University of Medical Sciences, Rafsanjan, Iran; ^2^Student Research Committee, School of Health, Rafsanjan University of Medical Sciences, Rafsanjan, Iran; ^3^Social Determinants of Health Research Center, Rafsanjan University of Medical Sciences, Rafsanjan, Iran; ^4^Non-communicable Diseases Research Center, Rafsanjan University of Medical Sciences, Rafsanjan, Iran

**Keywords:** COVID-19, happiness, health, spiritual, students

## Abstract

It is necessary to study the various dimensions of health and their affecting factors during the coronavirus disease-19 (COVID-19) pandemic to identify the necessary interventions. The study aims to determine the relationship between spiritual health and happiness in medical students during the COVID-19 outbreak. In this analytical cross-sectional design study, 409 medical students were examined for the state of happiness and spiritual health and the relationship between them. Student information was collected through Web-based sampling by using standard tools from 20 April to 20 June 2020. Medical students completed the demographic questionnaire as well as Oxford Happiness Questionnaire (OHQ) and Paloutzian and Ellison spiritual health questionnaire. The results showed that while the score of spiritual health and happiness was related to factors such as marriage, interest in a field of study, and socioeconomic status, the relationship between spiritual health and happiness was significant (*r* = 0.72). This study showed that students’ happiness scores were not optimal during the COVID-19 pandemic. Due to the strong relationship between spiritual health and happiness scores, spiritual health promotion, in conjunction with other interventions, can be used to improve happiness in this group.

## Introduction

According to the World Health Organization’s (WHO) definition of health, health is a multidimensional issue in which in addition to the physical, mental and social dimensions, the spiritual aspect is an integral and important part of health and also it should be noted that different dimensions of health are affected by each other (Chirico, 2016). Spiritual health is so important that studies have shown that without it, other biological, psychological, and social dimensions of health cannot function properly, and thus the highest level of quality of life cannot be achieved (Akbari and Hossaini, 2018). Spiritual health, which consists of two components: religious health (feeling of health and relationship with supernatural power) and existential health (relationship with others and the environment), is manifested by characteristics such as life stability, peace and harmony, a sense of closeness to oneself, a relationship with God, society, and the environment (Ebadi et al., 2017). Despite all the theoretical advances in the field of health, most health promotion programs ignore the spiritual dimension of health and the development of the concept of spiritual health is very slow and has been delayed in the field of health and disease prevention (Abbasian et al., 2016).

Spirituality is another human capability that provides problem-solving and coping strategies, as well as an increased sense of indirect control over events (Shah et al., 2011), and it is an important part of human life (Juškienė, 2016). Spiritual health is defined as a sense of meaning and purpose in life, as well as a connection to a higher power that enables people live better lives (Kamian, 2014). When people voluntarily reinforce their spiritual strengths through prayer, relaxation, communication with like-minded people, and learning from a spiritual guide and study, they achieve spiritual health (Carmody et al., 2008; Razaghi et al., 2019).

When a person’s spiritual health is seriously compromised, he or she may experience mental disorders such as loneliness, depression, and loss of meaning in life (Bonelli et al., 2012). Recent studies have shown that religious duties and interests have a significant and positive relationship with human health and longevity (Flannelly and Galek, 2010). Several other studies have also found an association between spirituality, better physical and mental health (VanderWeele et al., 2017). Strengthening one’s spiritual health improves one’s ability to adapt to changing circumstances. Spirituality also improves a person’s outlook on the world (Aramideh et al., 2018). Spirituality improves human mental health by providing social support and increasing self-efficacy and cohesion (Chen and VanderWeele, 2018). Happiness is one of the factors that influence spiritual health (Jalali et al., 2019). Spirituality is one of the most important factors in increasing happiness and public health. Moreover, people with higher religious beliefs have higher levels of happiness and health (Banisi, 2019). Happiness is a positive and pleasant emotional state associated with experiences such as life satisfaction (Varee et al., 2017). Some studies have shown that the spiritual dimension of health has an impact on happiness (Pandya, 2017). People who are unhappy are more likely to suffer from depression, anxiety, and its consequences, and they are more prone to addiction and abnormal social behaviors, as well as having a shorter life expectancy (Hosseini Kasnavieh et al., 2015). Therefore, it is necessary to focus on promoting spiritual health and happiness in youth, and this is especially important in students who are always exposed to a scientific and research environment (Mozafarinia et al., 2014). Iranian students face a lot of stress daily that compromises their mental and physical health. In such cases, spirituality is the only source that can help them (Ebadi et al., 2017) and promoting their spiritual health will play an effective role in improving mental health and coping with mental disorders (Murali et al., 2016).

The COVID-19 is currently causing one of the worst global crises in human history (Wang et al., 2020; Zakeri et al., 2021a). COVID-19 was discovered in China at the end of 2019 and quickly became a pandemic in the world (Rothan and Byrareddy, 2020). COVID-19 has an impact on physical health, individual, society and global psychosocial implications due to the increasing number of cases and deaths (Bo et al., 2020; Wang et al., 2020). With more and more reports, the COVID-19 virus in China has caused public panic and psychological health distress. Other countries are also experiencing COVID-19 problems (Ho et al., 2020). COVID-19 puts a lot of pressure on people, both physically and psychologically, culturally and socially, and has resulted in some behavioral and cultural changes such as home quarantine and social restrictions, in addition to physical problems (Sheivandi and Hasanvand, 2020; Zakeri et al., 2021b). It should be noted that both the epidemic diseases themselves and the strategies used to combat them have had negative psychological effects, and that a lack of attention of researchers to this area can lead to wider psychological damage (Shahyad and Mohammadi, 2020). Critical disease conditions can create new symptoms and disorders such as increased feelings of loneliness, decreased life expectancy, anxiety, and panic and reduced social support, all of which have negative psychological and social consequences that affect the mental health of society in some way (Hossini Rafsanjanipoor et al., 2021; Zakeri et al., 2021c). Several studies have found that the COVID-19 pandemic has had a devastating impact on various aspects of mental health (Olashore et al., 2021; Rajabimajd et al., 2021; Zakeri et al., 2021d). As the outbreak of COVID-19 has had a significant impact on students’ lives and academic activities, they are more likely to develop psychological symptoms during the outbreak of COVID-19 (Pramukti et al., 2020; Ahorsu et al., 2021; Nayan et al., 2022). When compared to students in other fields, the prevalence of mental health problems among medical students is constantly increasing (Stewart-Brown et al., 2000). During the outbreak of the COVID-19, many problems have been reported among medical students, such as perceived stress and anxiety (Sharma et al., 2021) and decreased sleep quality (Tahir et al., 2021). The students studying in the medicine-related programs are one of the groups at risk of impaired mental wellbeing, and this group has rarely been studied in terms of side effects such as decreased happiness (Arslan, 2021). Despite these complications, and due to the new nature of this pandemic, there is little information on how to prevent and control potential complications on people’s mental health status (Zakeri et al., 2021b). On the other hand, some studies show that spiritual health, as a meaning-based coping strategy, can be effective in reducing the stress of COVID-19 crisis and its effects on mental health (Arslan and Yıldırım, 2021). Therefore, if the level of happiness is low and is related to spiritual health, solutions can be proposed to increase the feeling of happiness in students, such as programs to promote spiritual health to improve the mental health of students during the COVID-19 pandemic.

In this regard, for better planning in various scientific fields, especially in the field of spiritual health and happiness of any society, happiness research is required (Duncan, 2010) to be aware of the current situation of that society in that field. Considering the importance of this issue and its role in student’s lives and their academic success, this study aimed to investigate the levels of spiritual health and happiness, as well as the relationship between them among students in COVID-19 crisis.

## Method

### Study design and setting

This was a descriptive-analytical study. The research settings were Rafsanjan University of Medical Sciences (RUMS), located at south east of Iran. Presently, the university benefits from four schools admitting students in a wide and varied range of fields of medicine, dentistry, nursing and midwifery, health and allied medical sciences.

### Sampling and sample size

The present descriptive study used the multistage random sampling method to evaluate the level of spiritual health and happiness and to determine the relationship between them.

Four faculties participated in the study. In the first stage, two class was randomly selected from the entrance of each field. Then, according to the list of students of each class, about half of the students were selected randomly and their consent to participate in the study was obtained. The inclusion criteria were students from all the semesters who had completed at least 6 months in medical college, students’ consent to participate in the study and the absence of a history of unfortunate events such as the death of a loved one or a serious illness in themselves and family members in the previous month. Participants with a history of mental disorders (Self-reported) and incomplete questionnaires were excluded from the study. The sample size was estimated to be 347 individuals using *p* = 0.5 and α = 0.05, *r* = 0.15 and the sample size formula, In order to increase the accuracy of the results and due to the possibility of dropping 20% of the samples, 415 people were included in the study, and the information of 409 people who were completely completed was evaluated.


(1) $$N=\left[\frac{\left(Z\,\alpha +Z\,\beta \right)}{\text{C}}\right]\,2+3=347$$

### Measures

Data were collected with three questionnaires: A: demographic information, B: the Ellison Spiritual Health Questionnaire, and C: the standard OHQ.

#### Demographic information questionnaire

it includes gender, marital status, economic status of the family, the field of study, interest in the field, housing, and coronavirus symptoms.

### Paloutzian and Ellison spiritual health questionnaire

The spiritual health scale of Paloutzian and Ellison (1991) consists of 20 items with three subscales: cognition, emotions, and action. The Paloutzian and Ellison Spiritual Health Questionnaire consists of 20 questions with response scales such as strongly agree, agree, neutral, disagree and strongly disagree on the Likert scale. Each option is assigned a score ranging from 1 to 5. The total score of spiritual health is the sum of the scores of the three dimensions of cognition, action, and emotions, which is between 20 and 100. Its validity and reliability have been confirmed (Alpha coefficient = 0.82) (Rahimi et al., 2014). This questionnaire has also been used by other Iranian researchers, and its reliability has been confirmed (Mozafarinia et al., 2014). In the present study, the Cronbach’s alpha for this questionnaire was 0.92.

### The Oxford happiness questionnaire

The OHQ with 29 questions was used to measure happiness. The OHQ was developed by Hills and Argyle (Hills and Argyle, 2002). The score range of this questionnaire is between 0 and 87 (each question with a 4-point Likert scale and a score between 0 and 3). The questionnaire is divided into five sections: life satisfaction, self-esteem, actual wellbeing, satisfaction, and positive moods. The reliability and validity of this questionnaire were measured by Alipour and Agah Heris (2007) and the results showed that it had good validity and reliability for measuring happiness in Iranian society. In the study of Jafarzadeh et al. (2015) the reliability coefficient of the scale was 0.84 using Cronbach’s alpha. In the present study, the Cronbach’s alpha for this questionnaire was 0.94. According to the cultural diversity, there is no cut-off point for classification. However, according to the maximum score that can be obtained and the average score obtained in order to better understand the subject and according to some similar studies, the happiness score is divided into high, low and moderate.

### Data collection

A computer expert assisted in the design of the electronic form of the demographic information questionnaire and other questionnaires. The research team tested and controlled this questionnaire in terms of efficiency and responsiveness. An online questionnaire was tested on 50 medical students to assess the involvement of individuals in completing the questionnaire. Due to the fact that the current research was conducted during the height of the COVID-19 crisis and the red state of COVID-19, it was not possible to complete the questionnaire in person. First, it was explained over the phone about the objectives of the project, the confidentiality of the information, and the questions and possible answers. Then, the students were asked to complete the questionnaires on the Internet within a period of about 10 min. During this period, classes were held virtually. Data were collected from 20 April to 20 June 2020.

### Data analysis

Frequency, percentage, mean and standard deviation were used to describe the sample characteristics. Descriptive statistics and one-sample *t*-test, analysis of variance and Pearson correlation were used to measure students’ spiritual health and its relationship with happiness. The normality of the data was checked and confirmed. The Kolmogorov-Smirnov test was used to ensure that the data were normal. Due to the quantitative nature of the variables, the Pearson correlation coefficient was used to check their relationship. The data were interpreted using SPSS 20 with a significance level of less than 0.05 was used to interpret the data.

### Ethical considerations

The ethics committee of Rafsanjan University of Medical Sciences approved the study protocol (IR.RUMS. REC.1399.104). An informed consent form was placed at the beginning of the electronic form. The objectives of the study, the confidentiality and anonymity of the information were explained in it.

## Result

The study included 409 students aged between 18 and 31 years old, with the mean age of 21.6 ± 2.3. The majority of the students were female (*n* = 293, 71.3%). Most of the students were health students (*n* = 104, 25.3%) with a medium socioeconomic status (*n* = 361, 87.8%). Only 11 participants (2.7%) reported COVID-19 symptoms during the study, and 59 patients (13.9%) reported COVID-19 symptoms in their close relatives. Table 1 shows the demographic characteristics of the students.

**TABLE 1:** Demographic variables of students and comparison of mean and standard deviation of happiness score and spiritual health in the study population (*n* = 409).

Variables	Frequency (valid percent)	Mean of spiritual health	*P*-value	Mean of happiness
**Gender**				
Man	116 (28.36)	72.98 ± 14.01	0.17	35.02 ± 17.39
Female	293 (71.63)	74.82 ± 11.85		33.82 ± 17.48
**Marital status**				
Married	58 (14.1)	81.87 ± 9.40	*P* < 0001*	43.86 ± 18.29
Single	351 (85.4)	73.05 ± 12.53		32.56 ± 16.79
**Residence**				
Native students	141 (34.8)	75.13 ± 12.78	0.32	36.81 ± 18.48
Non-native students	266 (64.7)	73.85 ± 12.37		32.73 ± 16.72
**College**				
Medicine	100 (24.3)	73.2 ± 13.2	0.14	38.32 ± 17
Paramedical	83 (20.2)	76.46 ± 12.9		35.20 ± 16.14
Nursing	73 (17.8)	72.8 ± 14.8		35.65 ± 18.24
Health	104 (25.3)	75.2 ± 11.57		34.08 ± 17.80
Dentistry	36 (8.8)	75.86 ± 12.04		33.00 ± 17.37
**The economic situation**				
Weak	27 (6.6)	67.96 ± 15.03	0.002*	26.55 ± 16.47
Medium	361 (87.8)	74.41 ± 12.14		34.16 ± 17.03
Good	21 (5.1)	80.52 ± 12.33		44.00 ± 21.25
**Interest in the field of study (Medicine)**				
Low	28 (6.8)	66.75 ± 13.40	*P* < 0001*	26.39 ± 15.52
Medium	176 (42.8)	71.73 ± 12.39		29.64 ± 14.65
High	205 (49.9)	77.54 ± 11.56		39.10 ± 18.49

* Significance level less than 0.05.

The mean score of spiritual health among students was 74.30 ± 12.51. Table 2 shows spiritual health scores and its dimensions in terms of demographic factors. The mean score of happiness among students was 34.16 ± 17.44. Table 1 indicates the happiness score and its dimensions in terms of demographic characteristics. Although spiritual health (*p* = 0.17) and happiness were not significantly different between the two genders (*p* = 0.53), happiness scores were significantly different according to marital status (*p* < 0001), housing (*p* = 0.024), economic status (*p* = 0.003) and interest in the field of study (*p* < 0001). Spiritual health scores were significantly different according to marital status (*p* < 0001), economic status (*p* = 0.002) and interest in the field of study (*p* < 0001) (Table 1).

**TABLE 2:** Mean and standard deviation of students’ spiritual health and happiness dimensions (*n* = 409).

Dimensions	Mean and standard deviation	Range
Cognitive dimension (SH)	23.98 ± 4.14	6–30
Emotions domain (SH)	30.83 ± 6.36	9–45
Action dimension (SH)	19.48 ± 3.46	5–25
Spiritual Health	74.30 ± 12.51	27–100
Life satisfaction (H)	10.50 ± 5.27	0–32
Self-esteem (H)	7.74 ± 4.87	0–28
Subjective wellbeing (H)	4.06 ± 3.45	0–20
Self –Satisfaction (H)	5.50 ± 2.71	0–16
Positive mood (H)	6.37 ± 3.36	0–20
Happiness	34.16 ± 17.44	0–85

SH, spiritual health; H, happiness.

The results of the correlation coefficient test showed that there was a statistically significant and positive relationship between students’ spiritual health score and happiness, as the score of spiritual health increases, so does the score of happiness. The results also showed a statistically significant relationship between all dimensions of spiritual health and happiness, although the strength of this relationship was variable (Table 3).

**TABLE 3:** Correlation coefficient of spiritual health and happiness dimensions in students (*n* = 409).

Variable	1	2	3	4	5	6	7	8	9
1–Cognition (SH)	1								
2–Emotions (SH)	0.69**	1							
3–Action (SH)	0.67**	0.70**	1						
4–Total spiritual health	0.87**	0.93**	0.85**	1					
5–Life Satisfaction (H)	0.60**	0.81**	0.60**	0.77**	1				
6–self- esteem (H)	0.45**	0.67**	0.49**	0.63**	0.79**	1			
7–Subjective wellbeing (H)	0.34**	0.55**	0.41**	0.51**	0.67**	0.78**	1		
8–Self-Satisfaction (H)	0.51**	0.62**	0.48**	0.62**	0.71**	0.72**	0.67**	1	
9–Positive mood (H)	0.45**	0.60**	0.48**	0.59**	0.72**	0.73**	0.68**	0.71**	1
10–Total happiness	0.54**	0.75**	0.57**	0.72**	0.77**	0.63**	0.51**	0.62**	0.59**

SH, spiritual health; H, happiness. ** Significance level less than 0.001.

Multiple regression models were tested to explore how demographic variables, spiritual health, and happiness can all be used to predict spiritual health and happiness. As shown in Table 4, happiness, marital status, interest in the field and economic status of the family all predict 53% of the variance of spiritual health (*R*^2^ = 53%), with happiness being the best predictor (*p* < 0.001). Spiritual health, gender, interest in the field and housing all predict 53% of the variance in happiness (*R*^2^ = 53%), with spiritual health being the best predictor (*p* < 0.001).

**TABLE 4:** Multiple regression analysis summary for underlying variables of spiritual health and happiness among students (*n* = 409).

Variable		β	P	95% CI Lower	95% CI Upper	R^2^
Spiritual health	Constant	–	<0.001	42.43	54.36	53%
	Happiness	0.66	<0.001	0.42	0.53	
	Marital status	0.09	0.008	0.84	5.75	
	Interest in the field	0.08	0.017	0.30	3.10	
	Economic status of the family	0.06	0.049	0.00	4.96	
Happiness	Constant	–	<0.001	–56.56	–40.43	53%
	Spiritual health	0.70	<0.001	0.88	1.07	
	Gender	0.07	0.020	0.48	5.63	
	Interest in the field	0.07	0.030	0.21	4.11	
	Housing	0.07	0.028	0.29	5.16	

Data were presented as multiple regression analysis. Only significant results were shown. CI, Confidence intervals for B.

## Discussion

This study aimed to evaluate the state of happiness and its determinants in students during the coronavirus epidemic. The results of the happiness study showed that the happiness score was low in this group. In different studies conducted on medical students in recent years to assess happiness, the mean happiness score ranged from 42.6 to 46.7 and was at a moderate level (Jouybari et al., 2017; Sadeghi et al., 2019). In this regard, the results of current study have been different from those of many other studies that have examined happiness in students, even though the mean score of happiness in medical students in the Sahraian study was higher than in the present study (Sahraian and Vakili, 2012). According to Kamthan et al. (2019), 60.8% of medical students were in the happiness group, and the happy population was made up of people who scored high on the happiness scale. To explain this difference, it should be noted that in the present study, happiness was measured during the coronavirus epidemic. Decreased emotional satisfaction and happiness can be expected because of the severity and scope of the coronavirus crisis. In this regard, studies conducted during the COVID-19 crisis revealed that the epidemic had a significant impact on emotional satisfaction and happiness. According to Yang and Ma (2020) the onset of coronavirus disease in China led to a 74% reduction in the feeling of living happily. Furthermore, Brodeur et al. (2020) showed that the COVID-19 epidemic had a negative effect on wellbeing and mental health and increased the feeling of sadness.

Therefore, it seems that students’ happiness and mental health will suffer as a result of their fear of COVID-19. Numerous studies have suggested that the COVID-19 crisis is responsible for psychological disorders (Holmes et al., 2020; Hossini Rafsanjanipoor et al., 2021; Zakeri et al., 2021e). Some studies also showed that happiness, in addition to having a positive effect on physical and mental health, has a negative effect on students’ academic achievement (Tabbodi et al., 2015). Therefore, strategies to raise the level of hope for the future and increase the feeling of happiness will be helpful in this situation. There are, of course, studies that show that some societies, even during COVID-19 crisis, cited high happiness, which could be due to the difference in the happiness level in different societies (Paz et al., 2022).

In the present study, the lowest score in the domain of happiness was related to the dimension of actual wellbeing and the highest score was related to satisfaction, which is consistent with the study of Mozafarinia et al. (2014). Daily activity restrictions seem to cause students to report low levels of vitality and happiness. Happiness was not significantly different between the two sexes in this study, but marriage, interest in the field of study, appropriate economic level and indigenousness were all positively associated with happiness. In line with the present study, some previous studies have shown no relationship between happiness and gender (Siamian et al., 2012).

Some studies have reported contradictory results. Calderon et al. (2019) showed that female students had higher happiness scores. This difference could be attributed to a variety of factors. Studies on students show the effect of social and economic factors on happiness. Furthermore, some studies show the effects of cultural diversity on happiness (Tuntiwarodom and Potipiti, 2008). Therefore, these differences may affect the difference in happiness between the two sexes in different studies.

In the present study, the level of spiritual health was moderate, which is consistent with many studies conducted on students (Dastgheib et al., 2015). In the present study, there was no difference in spiritual health scores between the sexes, while married students had better spiritual health. Although some studies, such as the Abbasi study, found no significant relationship between demographic variables and spiritual health (Abbasi et al., 2014), other studies have found that spiritual health is higher in female students (Tavan et al., 2015; Hasanshahi et al., 2016), which contradicts with the results of the present study. Although the score of spiritual health was higher in females, there was no statistically significant difference. Perhaps, the different conditions of the study and the existence of the COVID-19 disease crisis can be mentioned in justifying this difference, and more studies on spiritual health in crisis in both sexes are suggested. In the present study, the emotional domain of spiritual health received a higher score, which is consistent with the study of Ebadi et al. (2017).

The main purpose of this study was to investigate the relationship between spiritual health and happiness. The results of this study showed a relationship between all dimensions of spiritual health and happiness. Some research shows a positive relationship between religiosity and spirituality and various aspects of wellbeing, including physical health, mental health, life satisfaction and happiness (VanderWeele, 2017).

The results of this study are consistent with the results of other studies in this field (Mozafarinia et al., 2014; Arani and Hamzeei, 2018). However, the correlation between spiritual health and happiness scores was higher in current study. Perhaps the higher coefficient can be justified by pointing out that the level of happiness is lower in the conditions of COVID-19 disease and according to Islamic culture, more emphasis is placed on spirituality to create calmness and cure in the conditions of COVID-19 disease. Numerous studies have emphasized the importance of promoting spiritual health during the crisis of COVID-19 disease in various ways (Koenig, 2020; Rathakrishnan et al., 2021). A study conducted in the faith community of United States during COVID-19 pandemic showed that the decline in religious activities was associated with a decrease in happiness in this group (Jacobi et al., 2022).

Spiritual health improves human mental health by providing social support and increasing self-efficacy and cohesion (George et al., 2002). Some studies have shown that promoting spiritual health can improve students’ sense of pleasure, happiness and mental health (Ahmadi foroushani et al., 2013). On the other hand, Some studies show that in the pandemic crisis, some parts of spiritual health, including the participation in social religious activities, are faced with challenges (VanderWeele et al., 2016).

However, there are conflicting studies in this field, such as the study of Ahmadi Foroushani and Yazd khasti (2013) which found no significant relationship between religious beliefs, depression and mental health. To justify this difference, it is worth noting that both religious and spiritual dimensions of spiritual health were measured in our study, while Ahmadi’s study only looked at religious beliefs and practices (Ahmadi Foroushani and Yazd khasti, 2013). However, according to the results of the present study and most studies conducted in this field, it seems that by emphasizing the promotion of spiritual health as one of the methods to improve peace, reduce frustration and increase happiness and joy in students, steps were taken in this stressful conditions for the community and medical students, who are responsible for promoting the community health. According to some studies, the strategy of spirituality was a practical and appropriate solution to resolve students’ anxiety (Chaves et al., 2015).

As mentioned, during the COVID-19 epidemic, students’ happiness has decreased and spiritual health was related to happiness. Meanwhile, happiness and spiritual health can affect mental health. Numerous studies show that spirituality and religiosity are correlated through behavioral, psychological, physiological and social factors that can affect people’s mental health. From a behavioral perspective, people with higher spirituality and religiosity are often committed to a healthier physical and mental lifestyle. All of them are directly related to happiness through controlling your mind (Amirian and Fazilat-Pour, 2016; Zimmer et al., 2016; Dehghan et al., 2021a). People in cultural and religious fields are happy and hope for a heavenly life. Because spirituality and religiosity help them to overcome adversities and tensions throughout life (Dehghan et al., 2021b). Participating in religious practices and paying attention to spirituality can be a means to relax people, which can promote mental health outcomes such as happiness (Leung and Pong, 2021). From a social perspective, frequent attendance at religious services positively affects happiness. Because it can lead to more social support by increasing intimacy and communication with others (Childs, 2010; Amirian and Fazilat-Pour, 2016), and make people happy and improve people’s mental health. Finally, more studies are needed to better understand the mechanisms that link happiness and spirituality to people’s mental health.

As this is one of the first studies to examine happiness in the COVID-19 disease, these results can be used to help plan for better health in various dimensions. However, this study had some limitations, including the fact that due to lack of access to students, questionnaires were completed virtually, which may have affected the accuracy of completion. It also seems that designing a native tool based on Islamic beliefs to measure spiritual health can create more accurate results regarding students’ spiritual health status. However, it is suggested that more reliable tools be used in future studies to evaluate happiness. In the present study, students participated from different disciplines. Although it was tried to avoid selection biases by random selection as much as possible, given that a number of disciplines were less participated, the results may not be generalized to all students.

## Conclusion

The results of this study showed that during the COVID-19 epidemic, happiness scores of students were low in general and across all dimensions. The results of the correlation test showed a positive relationship between spiritual health score and happiness dimensions.

According to the results of the present study and some similar studies, it seems that steps can be taken to improve the level of happiness in students by strengthening the dimensions of spiritual health during crises such as COVID-19. Additional interventional studies are suggested in this field.

## Data availability statement

The original contributions presented in this study are included in the article/supplementary material, further inquiries can be directed to the corresponding author.

## Ethics statement

The studies involving human participants were reviewed and approved by the Rafsanjan University of Medical Sciences. The patients/participants provided their written informed consent to participate in this study.

## Author contributions

MA, MM, and SL: concept and design. MM and SL: data collection. MA and MZ: drafting of the manuscript, critical revision of the manuscript, and statistical analysis. All authors contributed to the article and approved the submitted version.
